# Investigation of optimizing indocyanine green solution for in vivo lymphatic research using near-infrared fluorescence indocyanine green lymphangiography

**DOI:** 10.1038/s41598-023-40826-x

**Published:** 2023-09-11

**Authors:** Hwayeong Cheon, Sang Ah Kim, Bumchul Kim, Jae Yong Jeon

**Affiliations:** 1 Biomedical Engineering Research Center, Asan Medical Center, 88, Olympic-ro 43-gil, Songpa-gu, Seoul, 05505 Republic of Korea; 2 Department of Rehabilitation Medicine, Asan Medical Center, University of Ulsan College of Medicine, 88, Olympic-ro 43-gil, Songpa-gu, Seoul, 05505 Republic of Korea

**Keywords:** Preclinical research, Translational research, Medical imaging, Imaging and sensing, Circulation, Fluorescence imaging

## Abstract

Despite the tireless efforts of many researchers in lymphatic research, indocyanine green (ICG) solution conditions suitable for lymphatic circulation tests have not been perfectly established yet. We aimed to investigate the optimal in vivo conditions of ICG solution to avoid photobleaching and quenching effects, which may affect the accuracy of lymphatic circulation evaluation. After ICG fluorescence intensity (or ICG intensity) was assessed under different in vitro conditions, the image quality of brachial lymph nodes (LNs) and collecting lymphatic vessels (LVs) in eight rats was investigated. The in vitro results showed that ICG intensity depends on concentration and time in various solvents; however, the brightest intensity was observed at a concentration of 8–30 μg/mL in all solvents. ICG concentration in the albumin (bovine serum albumin; BSA) solution and rat’s plasma showed more than two times higher fluorescence intensity than in distilled water (DW) in the same range. However, saline reduced the intensity by almost half compared to DW. In the in vivo experiment, we obtained relatively high-quality images of the LNs and LVs using ICG in the BSA solution. Even at low concentrations, the result in the BSA solution was comparable to those obtained from high-concentration solutions commonly used in conventional circulation tests. This study provides valuable information about the conditions for optimal ICG intensity in near infrared fluorescence indocyanine green (NIRF-ICG) lymphangiography, which may be useful not only for the diagnosis of lymphatic circulation diseases such as lymphedema but also for preclinical research for the lymphatic system.

## Introduction

Near-infrared fluorescence lymphangiography or lymphography using indocyanine green dye (NIRF-ICG lymphangiography) is a primary technique in lymphatic research and clinical practice for diagnosis of lymphatic diseases^1–3^. In this minimally invasive imaging technique, indocyanine green (ICG), a fluorescent cyanine dye, is injected intradermally, and the flow and distribution of this dye in the body are analyzed using a near-infrared optical system. Further, because ICG molecules strongly bind to plasma proteins within the blood vessels and lymphatic vessels (LVs), they move along the blood and lymph fluid with few leakages out of the vessels^4,5^. Regarding optical properties, ICG molecules absorb infrared light between 600 and 900 nm, which has a 789 nm peak, and emit fluorescent light between 750 and 950 nm, which has an 814 nm peak^6^. Although the absorption and emission spectrum of ICG has overlapped largely, the superficial flow of the vascular and lymphatic systems in the body can be effectively visualized by controlling the absorption and emission window. Since ICG was approved by the FDA in 1959, it has played a crucial role in cardiovascular disease research and diagnosis. However, the use of ICG for lymphatic evaluation has only begun to gain attention relatively recently. Moreover, as it was used as an imaging technique to guide lymphadenectomy, also known as lymph node (LN) dissection, the potential application of NIRF-ICG imaging techniques for the lymphatic system has been suggested. Currently, NIRF-ICG lymphangiography has been established as a primary modality for lymphatic circulation disorders, especially lymphedema (LE).

NIRF-ICG lymphangiography provides (a) the presence of dermal backflow, which refers to leakage and accumulation of lymphatic fluid in the dermis and soft tissues by lymphatic disruption, (b) the anatomical location of LNs and LVs, and (c) the function of the LVs including lymphatic contraction and transfer capacity of the lymphatic fluid. Observing superficial lymphatic drainage in real time and evaluating the severity of LE is important for deciding the appropriate treatment methods. According to recent research, LE staging using NIRF-ICG lymphangiography is more useful for patients with breast cancer-related LE than the International Society of Lymphology (ISL) staging system, which is the clinical conventional gold standard ^2^. Moreover, Mihara et al. reported that NIRF-ICG lymphangiography is superior to other diagnostic modalities for LE, such as lymphoscintigraphy and computed tomography (CT) in clinical practice^7,8^.

Despite these advantages, studies for NIRF-ICG lymphangiography are still lacking in both preclinical and clinical research, and there is inadequate information regarding the optimal conditions of ICG solution required to obtain effective NIRF-ICG images or signals. In this study, we investigated the condition of ICG fluorescence intensity (or ICG intensity) to avoid the photobleaching and quenching effect of ICG fluorescence in various solutions and presented them for better visibility using the rainbow colormaps (Suppl. Fig. 1). In addition, based on the in vitro conditions, we examined ICG intensity under different conditions in the in vivo LNs and LVs of animal models.

## Results

### ICG fluorescence intensity depended on concentration and time in various solvents

**Figure 1:**
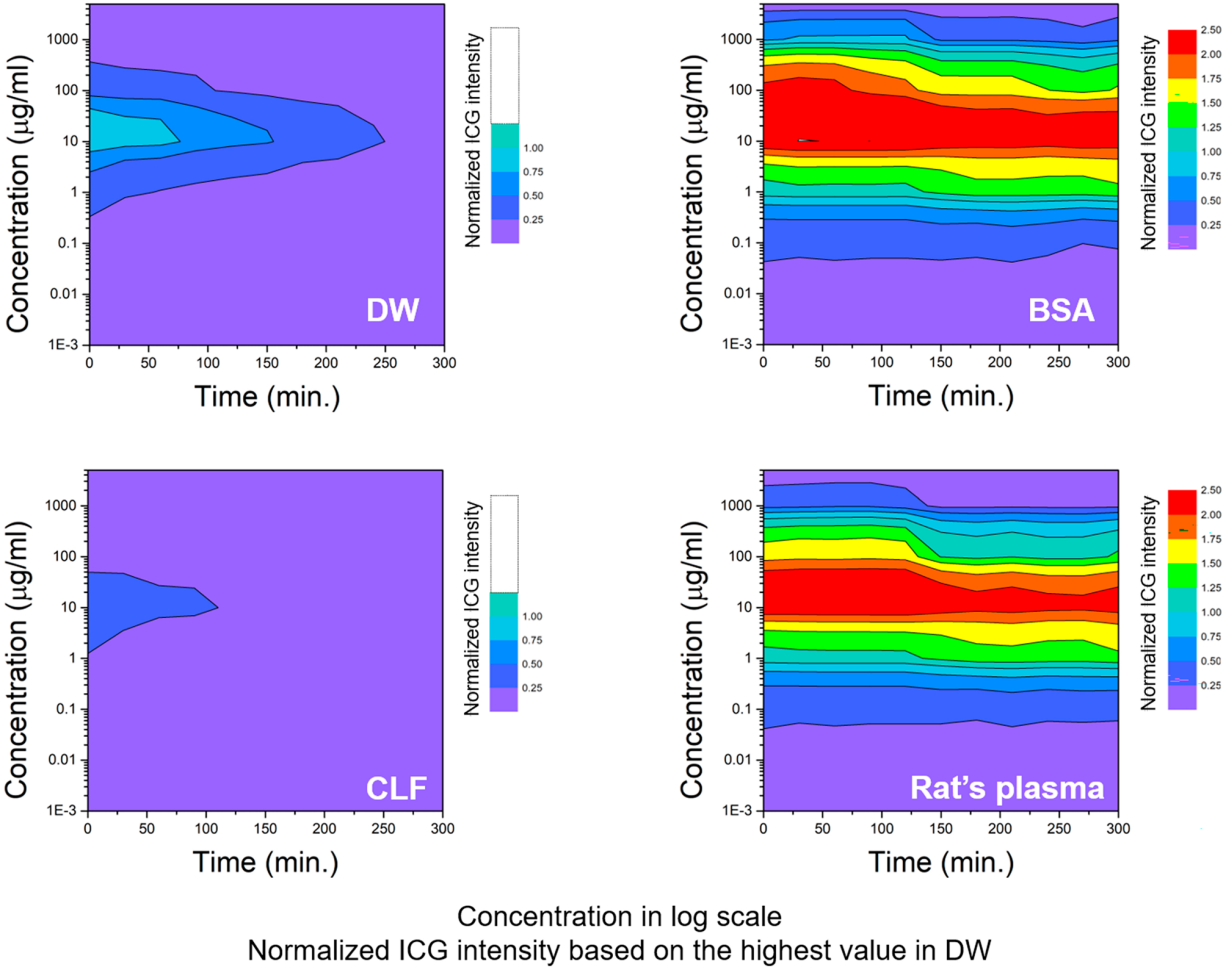
The rainbow colormap of indocyanine green (ICG) fluorescence intensity (ICG intensity) change (red: high ↔ purple: row) with the concentration and time in each solvent (DW; distilled water, BSA; bovine serum albumin solution of 25 mg/mL, CLF; commercial artificial lymphatic fluid, and rat’s plasma; separated plasma from whole blood of Sprague–Dawley rat). The highest value of DW was set to 1, and the rat's plasma and BSA solution showed more than two times brighter ICG intensity based on the highest DW value. The result values were determined by averaging values measured three times in six repeated experiments. Averaged values of standard deviation were 0.0619 (DW), 0.0170 (BSA), 0.0163 (rat’s plasma), and 0.0430 (CLF), respectively.

The results of all in vitro experiments were represented as relative values, with the highest brightness in distilled water (DW) set as a reference point and assigned a value of 1. Figure 1 shows the rainbow colormap of ICG intensity according to ICG contrition and measuring time in DW, bovine serum albumin (BSA) solution, Sprague–Dawley (SD) rat’s plasma, and commercial artificial lymphatic fluid (CLF). In the DW results, the brightest intensity (relative value of 1) was observed at a concentration of approximately 8‒30 μg/mL, and the intensity decreased at concentrations higher or lower than this range. In addition, the intensity gradually decreased over time. ICG showed more than two times higher fluorescence intensity in the BSA solution than in DW at a similar concentration range and maintained the brightness without significant decay over the 300-min measurement period. The overall trends of ICG intensity in the BSA solution were similar to those measured in the rat’s plasma sample, where ICG intensity also remained higher than that of DW over the measurement period. In contrast, CLF showed only about half the values compared of the ICG intensity in DW.

**Figure 2:**
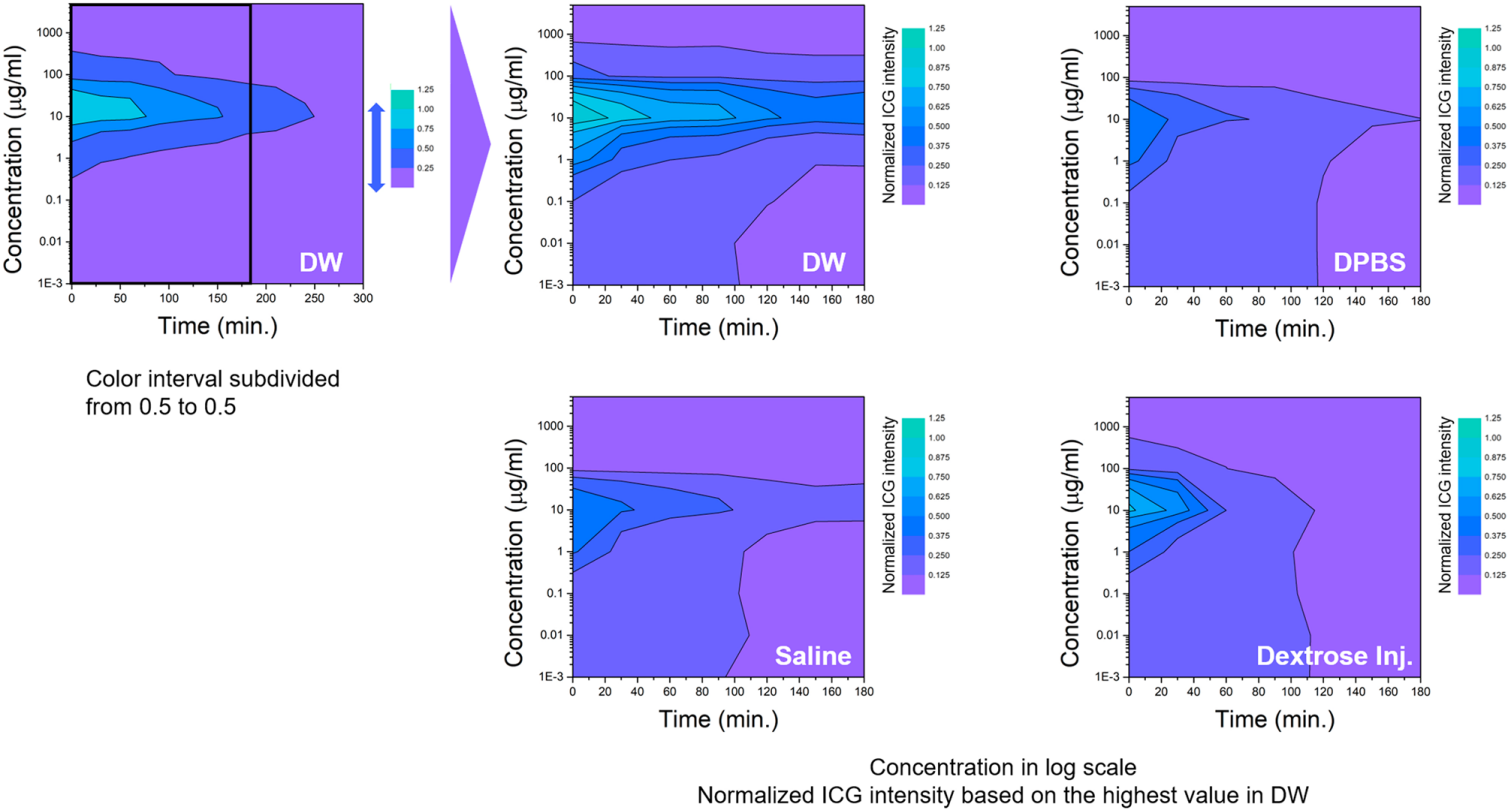
To analyze the change in ICG intensity in other solvents, we compared the rainbow colormap of ICG intensity change (cyan: high ↔ purple: low) with the concentration and time in various solvents such as DPBS (Dulbecco's phosphate buffered saline), saline, and dextrose injection solution based on the results of DW. We set the highest value of DW as 1 and further subdivided the color map because no value exceeded 1 in the result. However, the ICG intensity was only measured up to 180 min as the values rapidly decreased in most solvents, compared to that of Fig. 1. The result values were determined by averaging values measured three times in six repeated experiments. The averaged values of standard deviation were 0.0551 (DW), 0.0571 (DPBS), 0.0404 (dextrose inj.), and 0.0435 (saline), respectively.

The results of the injection solvents for ICG also were compared with the DW results (Fig. 2). Saline, one of the most commonly used solvents for ICG injection, reduced ICG intensity by almost half compared to DW, and the result was similar in the Dulbecco's phosphate buffered saline (DPBS) solution. The ICG intensity was slightly higher in the dextrose injection solution than in saline and DPBS solutions but decayed faster than in both solvents over time.

**Figure 3:**
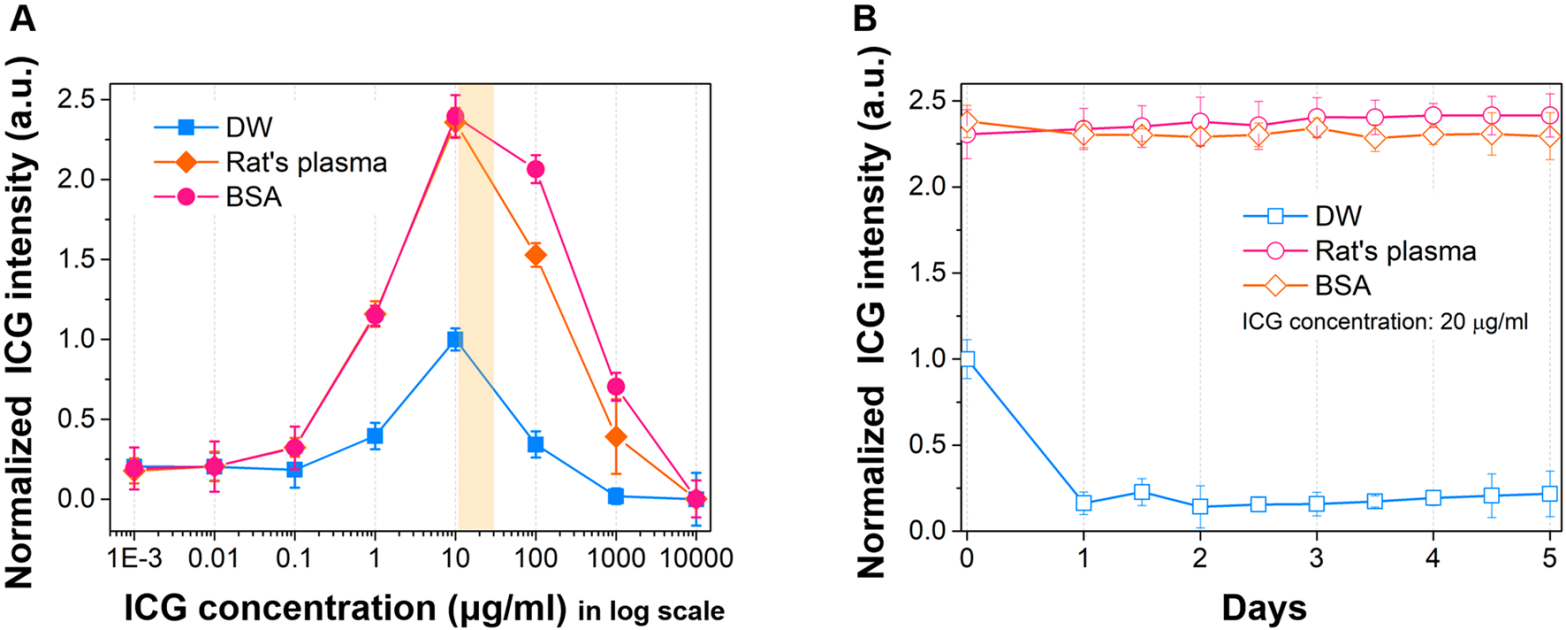
(**A**) ICG intensity in arbitrary units plotted against ICG concentration in DW, rat’s plasma, and BSA solution. (**B**) Normalized ICG intensity in arbitrary units over the days. The data was obtained by averaging repeated three measurements.

An additional measurement in DW, rat's plasma, and BSA solution was performed to compare the ICG intensity profile by the quenching and photobleaching effect in detail. Figure 3A shows the normalized ICG intensity with measurements performed immediately after the complete dissolution of ICG in each solvent as per the ICG concentration. The measured values were normalized according to the highest value obtained in DW. At approximately 20 μg/mL, all samples exhibited the highest fluorescence intensity, with ICG in rat's plasma and BSA solution presenting significantly higher intensity than in DW and approximately the same intensity as each other (almost 2.3-fold). Furthermore, regarding the photobleaching effect in each solvent, ICG molecules in rat's plasma and BSA solution continued to emit fluorescence for more than 5 days, while in DW, the fluorescence intensity decreased significantly after just 1 day (Fig. 3B).

### The ICG fluorescence spectrum in various conditions

**Figure 4:**
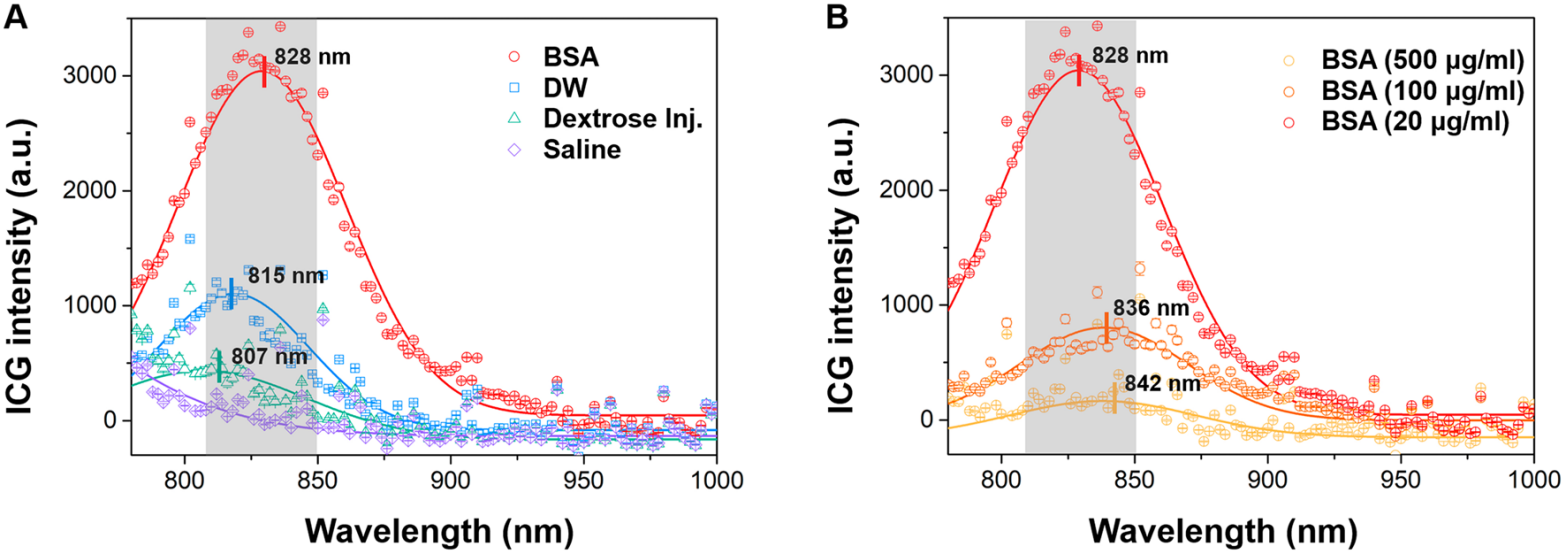
The spectral characteristics of ICG in various solvent conditions. The line graphs represent the fitting results of each data. (**A**) ICG fluorescence spectrum in different solvents of the same ICG concentration (20 μg/mL). (**B**) ICG fluorescence spectrum in each BSA solution of different ICG concentrations (500, 100, 20 μg/mL). The values were derived from averaged three repeated measurements. The gray area is the spectral window of the bandpass filter in our imaging system.

We also assessed the characteristics of the ICG fluorescence spectrum in various conditions using a spectrometer. The center of the peak of the ICG fluorescence spectrum was slightly blue-shifted in different solvents. The shift was correlated with the attenuation characteristics of each solvent, with the most significant blue shifts observed in the dextrose injection solution. No peak was observed in saline because it was difficult to identify peaks due to the low ICG intensity (Fig. 4A). We further observed the characteristics of the ICG fluorescence spectrum at each concentration in the BSA solution, which exhibited the highest intensity among all previously measured samples. As shown in Fig. 4B, the spectrum peak in the BSA solution shifted slightly to a longer wavelength (red-shift) as the concentration of ICG increased. However, the ICG fluorescence intensity decreased as the concentration increased due to the quenching effect.

### ICG fluorescence intensity differed in various solvents in the in vivo lymphatics

**Figure 5:**
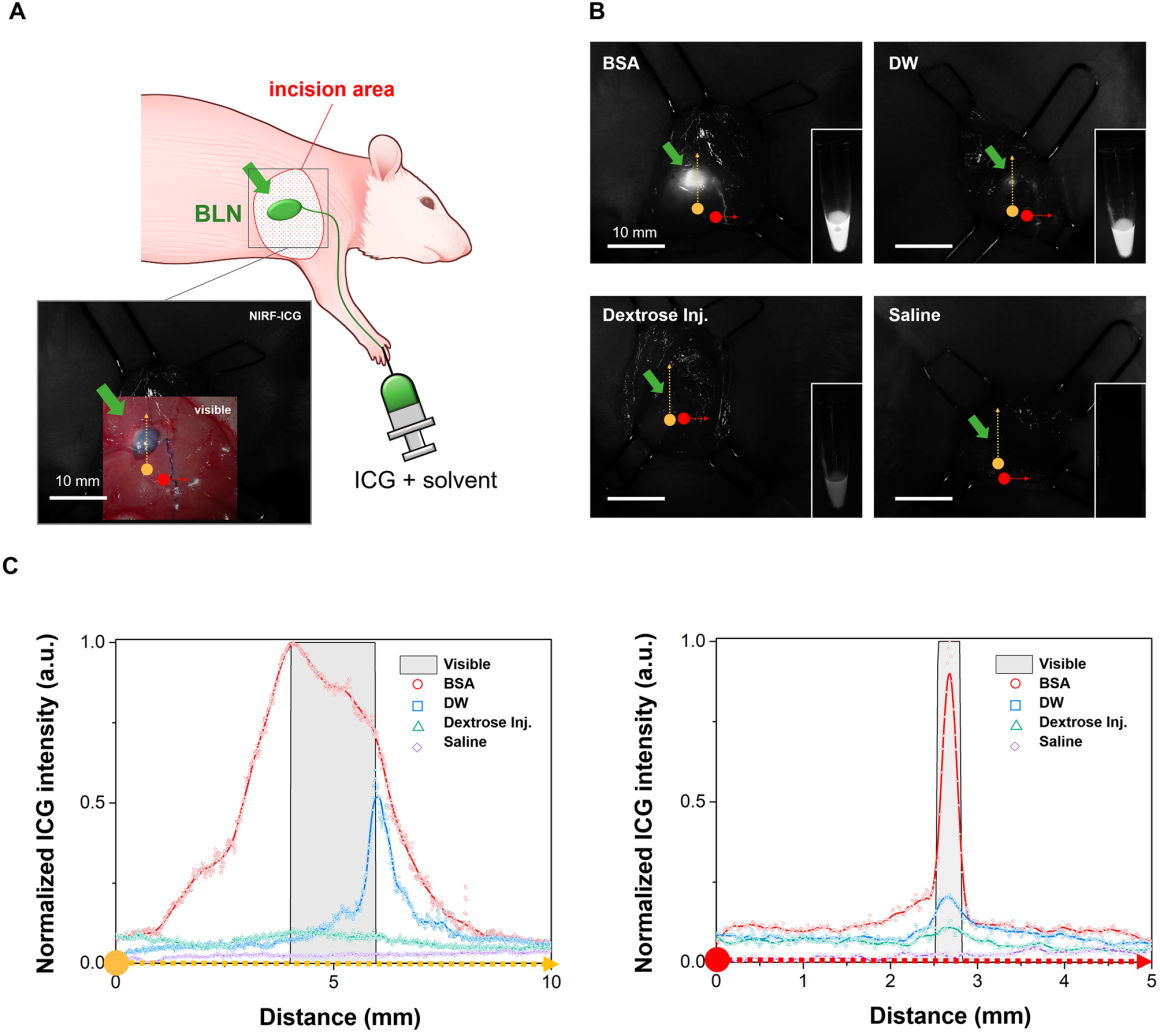
In vivo ICG fluorescence of BSA, DW, dextrose injection, and saline with the same ICG concentration (20 μg/mL) in the lymphatic system. (**A**) Incision area for observation of brachial lymph nodes (LNs) and collecting lymphatic vessels (LVs) connected to them. (**B**) ICG fluorescence images after injection of each ICG solution (inset). (**C**) The normalized ICG intensity of the cross-sectional profile of LNs (yellow dot lines in **B**) and LVs (red dot lines in **B**). The gray area graphs were derived from the visible images of the LNs and LVs verified using Evans blue injection (Suppl. Fig. 2B). The outcome values were derived by taking the average of three measurements (n = 4).

The characteristics of ICG intensity in BSA solution, DW, dextrose injection solution, and saline solvents were identified in vitro, and the quality of the in vivo fluorescence imaging was quantitatively compared by injecting ICG solution into the lymphatics (LNs and LVs) of small animals. The concentration was set to 20 μg/mL, which was the maximum intensity in all solvents, and a single injection was made into the web space of the paw in the upper limbs (Fig. 5A). Thereafter, the injected ICG solution flowed into the lymphatic system through the lymphatic capillaries in the paw and moved along the collecting LVs that pass through the extensors of the forelimb and the triceps brachii, and connect to the brachial LNs. The brachial LNs, located between the posterior region of the triceps brachii and the anterior region of the latissimus dorsi, are the dominant LNs where most of the lymph fluid collected from the upper limb. In this experiment, the collecting LVs and brachial LNs were observed from the dorsal direction of the upper limb. Figure 5B presents the ICG fluorescence images of brachial LNs (green arrow) and collecting vessels after the injection. We compared the image quality by investigating the cross-sectional intensity of LNs (yellow dotted lines) and LVs (red dotted lines) in the images. As shown in the cross-sectional profile of Fig. 5C, clearer LNs and LV images were obtained from the result of the BSA solution that showed the highest ICG intensity in the in vitro experiment. The signal-to-noise (SNR) of LN images was 21.8 dB for the BSA solution, 17.5 dB for DW, 5.7 dB for the dextrose injection solution, and 1.3 dB for saline, based on the noise level in each image. Regarding the LV images, the SNR was 18.7 dB for the BSA solution, 6.7 dB for DW, 4.9 dB for the dextrose injection solution, and 4.9 dB for saline. Regardless of the SNR results in the ICG fluorescence images, there was different between the width of the LNs and LVs measured with blue dye due to light leakage (Suppl. Fig. 2).

**Figure 6:**
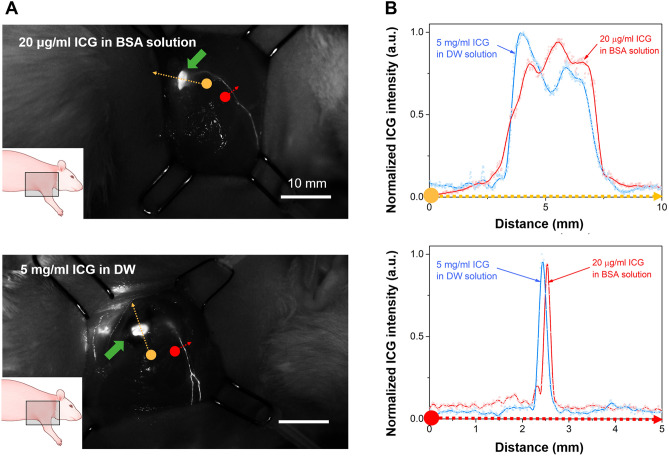
Comparison between 20 μg/mL ICG in BSA solution and 5 mg/mL ICG in DW. (**A**) In vivo images and (**B**) the cross-sectional spectrum of LNs and LVs. The outcome values were derived by taking the average of three measurements (n = 4).

The quality of fluorescence imaging obtained from the injection of 20-ug/mL ICG in BSA solution was also compared with the results obtained using the ICG injection concentration condition (5 mg/mL) commonly used in conventional blood circulation tests (angiography). In ICG angiography, a relatively high concentration of ICG is used so that it can be diluted in the bloodstream, and similar conditions are currently being used in lymphangiography. Figure 6 shows the fluorescence images and their cross-sectional intensities obtained by injecting the low ICG concentration (20 μg/mL) in the BSA solution and the high ICG concentration (5 mg/mL) in DW. The injection of the two solutions resulted in a comparable image quality, as evidenced by the similar cross-sectional intensity results in LNs and LVs.

## Discussion

NIRF-ICG lymphangiography is relatively simple to perform, minimally invasive, highly sensitive, involves no radiation, and accurately reflects the lymphatic system in real-time^9,10^. Therefore, NIRF-ICG lymphangiography has been used as a primary modality for lymphatic circulation diagnosis and lymphatic disease research in clinical practice and preclinical experiments. However, research on the optimal conditions of ICG solution for assessing lymphatic circulation is limited because NIRF-ICG imaging was mainly developed to examine liver function and vascular circulation. Indeed, most commercial ICG drug approved by the FDA, such as IC-GREEN® (Akorn), INDOCYANINE GREEN (Renew Pharm. Ltd), and SPY AGENT® GREEN (Novadaq Technologies Inc), provide guidance for their products primarily for angiography. Only the SPY AGENT® GREEN product offers separate information for visualizing the lymphatics, but its primary purpose is for lymphatic mapping in breast cancer treatment. Consequently, many researchers continue to conduct studies to assess lymphatic circulatory disorders related to LE and other related conditions^11–13^. Especially, Weiler et al*.*^13^ reported detailed ICG solution conditions for NIRF-ICG lymphangiography for the diagnosis of lymphatic diseases. They demonstrated that the SNR of ICG imaging in vivo significantly increased when albumin solution and ICG were used together. This approach has previously been proposed for sentinel LNs mapping in breast cancer treatment indicating that the albumin mixture can be used to enhance ICG intensity^14^. However, for effective optimization of NIRF-ICG lymphangiography to detect lymphatic disease, a comprehensive map of optimal conditions has been required. This map should consider not only the concentration that causes the quenching effect but also the time that induces photobleaching considering of the time it takes to prepare and inject the drug. Additionally, there is also a need for information covering both in-vitro and in-vivo regarding these optimal conditions.

In this study, we investigated the characteristics of ICG intensity based on various solvent conditions to determine the optimal ICG conditions for NIRF-ICG lymphangiography. In vitro experiments revealed that excessive ICG concentration in all solvents resulted in decreased ICG intensity due to a quenching effect^15–17^, and the concentration range that showed the highest fluorescence intensity was approximately 8–30 μg/mL. ICG aggregation occurred in the blue-shift zone of the emitted fluorescence spectrum^17^, and the increased photobleaching effect was attenuated over time. ICG stabilized by binding with albumin (in the BSA solution and rat’s plasma) and exhibited relatively lower photobleaching effects over a long period of time. However, solvents primarily used for injection, such as saline, increased ICG aggregation^9,18^, causing it to decay rapidly.

The results of the in vitro experiments on ICG intensity trends were consistent with those observed in the in vivo injection experiments, where ICG stabilized by binding with albumin and rapidly spread throughout the lymphatic system, producing clear lymphatic fluorescence images. Notably, the quality of fluorescence imaging achieved with low ICG concentrations in the BSA solution was comparable to that obtained with high ICG concentrations in DW. High ICG concentrations can increase the quenching effect in fluorescence, and the quenching effect of ICG causes a red-shift in the emitted fluorescence spectrum^19^, which may impede the efficiency of pre-set imaging devices. Additionally, using excessively high concentrations of ICG can increase the risk of toxicity^20^ and may interfere with accurately diagnosing lymphatic diseases. In the research of Weiler et al*.*, it was noted that high concentrations of ICG produce significantly large light leakage, which may interfere with visualizing lymphatics located under the skin. Additionally, Gashev et al*.* reported that lymphatic contraction and movement are able to be disturbed by ICG injection with high concentrations^21^. Considering that lymphatic contraction and flow speed are recognized as an indicator for lymphatic diseases in animals and humans^22–25^, it is important to use an appropriate concentration of ICG for accurate measurements. Thus, using high concentrations of ICG solution to obtain fluorescence imaging in clinical settings may negatively impact patient safety and image quality. Hence, this study results suggest that using lower ICG concentrations in combination with albumin solution may be a more suitable approach for lymphatic circulation rather than a high ICG concentration solution for vascular circulation tests.

There were some limitations of in vivo experiments. Although our study has demonstrated that using albumin solution allows for high quality image with lower concentrations of ICG, there may be disadvantages for long-term measurements due to the rapid clearance of the lower concentration ICG solution. This may be because the lower concentration solution contains fewer ICG molecules. We were unable to provide the information on how long the imaging could be sustained when using low concentrations of ICG in this experiment. Therefore, further research for NIRF-ICG lymphangiography is needed to determine how quickly ICG solutions with varying concentrations enter the lymphatic system, how long images of consistent quality can be acquired after the injection, and whether there are any differences depending on the injection sites.

## Methods

There were no statistical methods used to determine the sample size, and none of the samples were excluded from the analysis. All experiments were performed in accordance with the relevant guidelines and regulations of the Institutional Animal Care and Use Committee (IACUC) of the Asan Institute for Life Sciences, Asan Medical Center. The IACUC abides by the Institute of Laboratory Animal Resources (ILAR) and Animal Research: Reporting of In Vivo Experiments (ARRIVE) guidelines of The National Centre for the Replacement, Refinement and Reduction of Animals in Research (NC3Rs).

### NIRF imaging system and ICG solution

**Figure 7:**
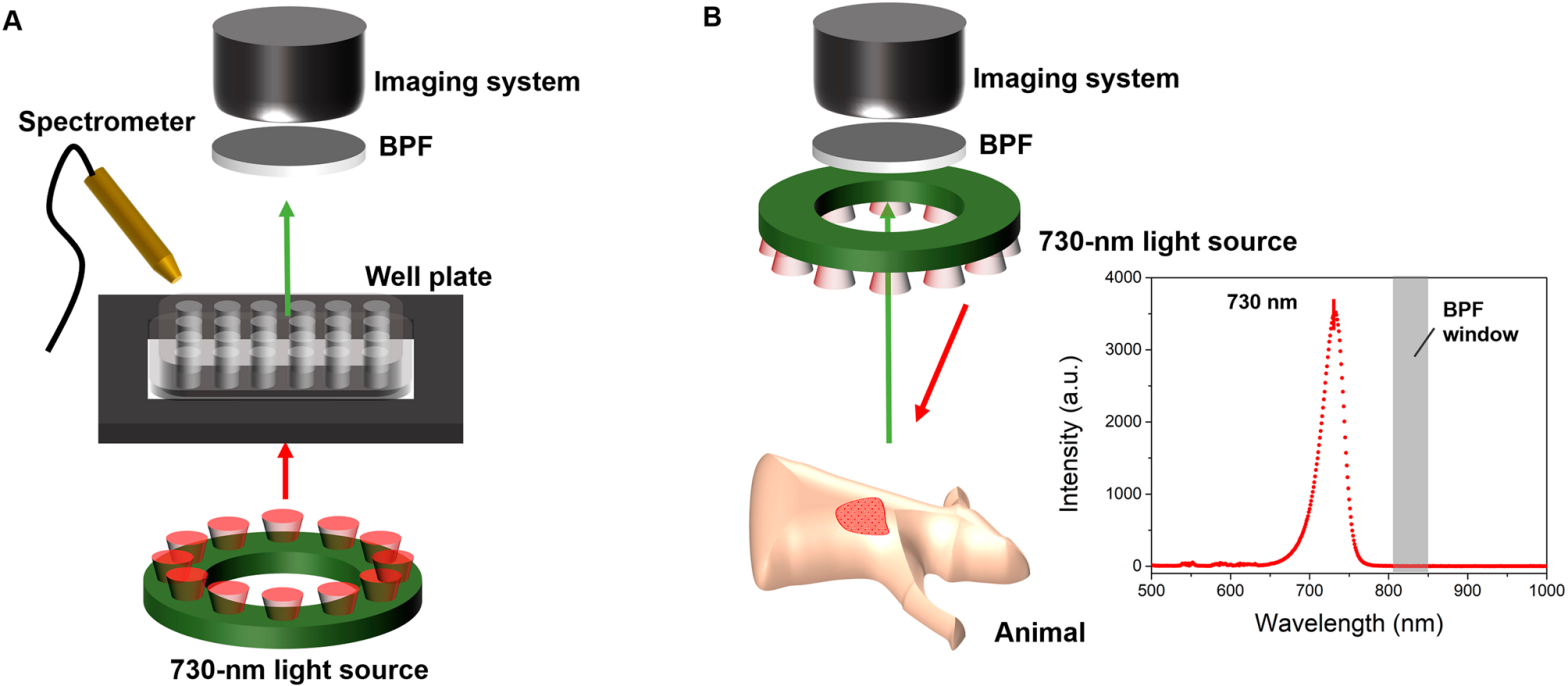
Schemes of the NIRF imaging system for (**A**) in vitro experiment and (B) in vivo experiment.

We used a near infrared fluorescense (NIRF) imaging system for both in vitro and in vivo experiments which was a customized device based on a commercial digital camera (Cannon EOS 50D; Cannon, Tokyo, Japan). We replaced the imaging sensor and adapted it for near-infrared measurements. The 2-inch bandpass filter (FF01-832/27-50-D; Semrock, West Henrietta, NY) that passes the light from a wavelength of 823 nm to 837 nm was attached in front of the lens. Twelve high-power LEDs of 730-nm peak (LST1-01G01-FRD1-00; Opulent Americas, Raleigh, NC) with a total output power of 4.2 watts were used to excite ICG fluorescence. In the in vitro experiment where we used transparent 96-well plates, the light source was installed under the well plate, and fluorescence was measured using the NIRF imaging system and a fiber optic spectrometer (USB-650 Red Tide Spectrometer; Ocean Insight, Orlando, FL) (Fig. 7A and Suppl. Fig. 3A). In the in vivo animal experiment, the light source was mounted in front of the lens and measured using the imaging system (Fig. 7B and Suppl. Fig. 3B). The images and spectrum were analyzed numerically and visualized using ImageJ 1.48v (NIH, Bethesda, MD) and Origin Pro 9 (version 9.0; OriginLab, Northampton, MA) software. The ICG powder was purchased from Daiichi-Sankyo company (Diagnogreen Injection 25 mg; Daiichi Sankyo co., LTD, Tokyo, Japan), and the solutions used in the experiment were prepared for each concentration immediately before measurement.

### In vitro experiments with the ICG solutions

**Figure 8:**
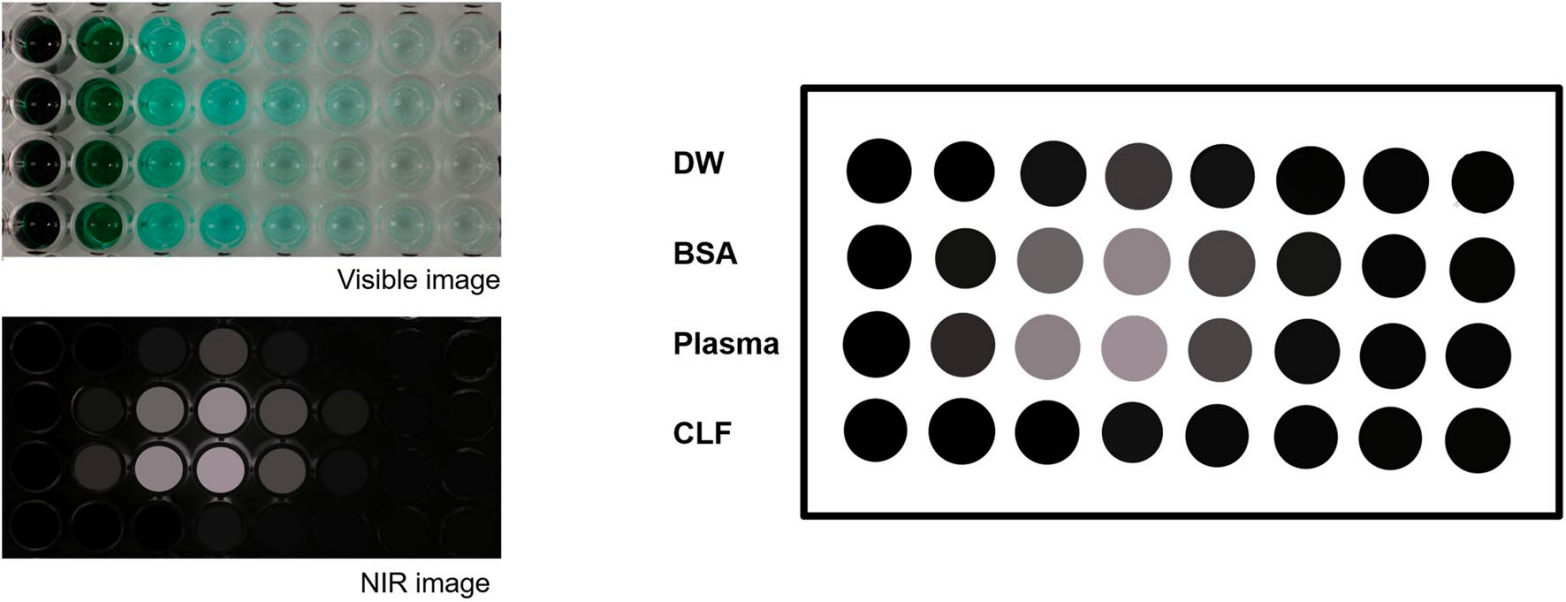
Measurement of ICG intensity in the 96-well plates for the in vitro experiment. The values of the ICG intensity in each well were averaged.

The ICG solutions used in the in vitro experiment were made by adding the experimental solvents to a 5 mg/mL solution (base concentration) and diluted to a concentration of 1,000, 100, 10, 1, 0.1, 0.01, and 0.001 μg/mL, respectively. We used DW, BSA solution (5 mg/mL; Sigma, St. Louis, MO), CLF (Simulated Lymph Fluid BZ267, CAS-No. 7732-18-5; Biochemazone, Alberta, Canada), rat’s plasma extracted from the animals, DPBS (Dubecco’s Phosphate Buffered Saline; Welgene Inc., Gyeongsan, Republic of Korea), saline (Normal Saline Injection; Dai Han Pharm CO., LTD., Seoul, Republic of Korea) and dextrose injection solutions (Dextrose Injection; 5%; Dai Han Pharm CO., LTD., Seoul, Republic of Korea) as solvents. The rat’s plasma was prepared by centrifugation (Eppendorf-5415R; Eppendorf, Hamburg, Germany) at 3000 RPM, 15 min from the whole blood sample of SD rats harvested from inferior vena cava and other commercial products. Each solution sample was put into the 96-well plates, 100 μL of concentration and solvent was added, and images and spectrum were measured (Fig. 8). Each experiment was measured six times, and the average of three values was taken for each measurement to determine the final result of ICG intensity.

### In vivo animal experiments

All animal procedures were approved by the Institutional Animal Care and Use Committee of the Asan Institute for Life Sciences, Asan Medical Center (Seoul, Republic of Korea). The surgical procedures for the animals were performed on specific premises with specifications. Eight male SD rats (12 weeks old, 400–450 g; Ja Bio, Suwon, Republic of Korea) were used for the experiments. The animals are randomly assigned to experimental groups of each injection without taking into account any additional factors or variables. All animal experiments were conducted by a single investigator, who used the same drug conditions as those employed in the *in-vitro* experiments for the animal experiments. Data collection was also performed by the same investigator. During the experiments, the rats were housed under constant temperature and humidity and were allowed to drink water and feed ad libitum freely. Prior to skin incision, the rats were anesthetized with tiletamine/zolazepam (50 mg/kg, Zoletil; Virbac, Carros, France) mixed with xylazine (volume ratio 5:1; Rumpun, Bayer Korea, Seoul, Republic of Korea). The fur in the upper limb was shaved and removed completely with electric clippers and depilatory cream after anesthetization because the fur scattered the excited and emitted light, making it difficult to obtain accurate results. To expose the subcutaneous tissue near the brachial LNs, the skin of the upper limb was incised 3–4 mm along the edge of the triceps brachii muscle and separated from the dermis layer. The surrounding fat tissue was removed, and the brachial LNs and collecting LVs were exposed in the subcutaneous tissue while taking care not to damage the LNs and LVs. After the procedure, 0.05-ml Evans blue solution (30 mg/ml solution in 0.9% saline; Sigma, St Louis, MO) was injection to identify the anatomical location of LNs and LVs in the image. Subsequently, each different 0.05 mL ICG solution was injected sub-dermally into the upper limb paw of each animal for the investigation of ICG intensity (1 μg as a single dose).

### Ethical approval

The number of animals and all animal procedures were approved and regulated by the Institutional Animal Care and Use Committee (IACUC) of the Asan Institute for Life Sciences, Asan Medical Center. The IACUC abides by the relevant guidelines including the ILAR and ARRIVE guidelines.

### Supplementary Information

Supplementary Information.

## Data Availability

All data generated or analysed during this study are included in this published article (and its Supplementary Information files).
