# Supplementary material for: Investigation of optimizing indocyanine green solution for in vivo lymphatic research using near-infrared fluorescence indocyanine green lymphangiography
Source: Sci Rep. 2023 Sep 11;13:14966. doi: 10.1038/s41598-023-40826-x (PMC10495419; doi:10.1038/s41598-023-40826-x)
Supplement: Supplementary file 1 — Supplementary Information. [file 41598_2023_40826_MOESM1_ESM.pdf]

Supplementary materials

Investigation of optimizing indocyanine green solution for *in vivo* lymphatic research using near-infrared fluorescence indocyanine green lymphangiography

The rainbow colormap for ICG intensity

In this study, we investigated the photobleaching (time dependance) and quenching (concentration dependance) effects of indocyanine green (ICG) molecules, which significantly affect imaging quality in near-infrared indocyanine green (NIRF-ICG) lymphangiography. The primary physical factors affecting photobleaching and quenching effects are measurement time and concentration of ICG molecules, respectively. Of course, they also are affected by other physical factors such as temperature and pressure, but the measurement time and concentration of solution are the most considerable factors in preclinical and clinical settings. It is better to visualize the ICG fluorescence intensity (ICG intensity) by measurement time and concentration of solution at the same time because the two factors can simultaneously affect the. The rainbow colormap is an effective plotting method for visualizing the effect of two variables in a two-dimensional space using color. We prepared ICG solutions of varying concentrations in different solvents and simultaneously measured the ICG intensity at 30-minute intervals to obtain two-dimensional matrix data. As shown in Supplementary Figure 1, the horizontal and vertical axis data of the 2D colormap represent the temporal change in ICG intensity at a fixed concentration and the change in ICG intensity depending on the concentration at a certain time, respectively. In Figure 1 and Figure 3, the optimal conditions for ICG intensity in various solvents by these two variables can be obtained by using these colormaps.

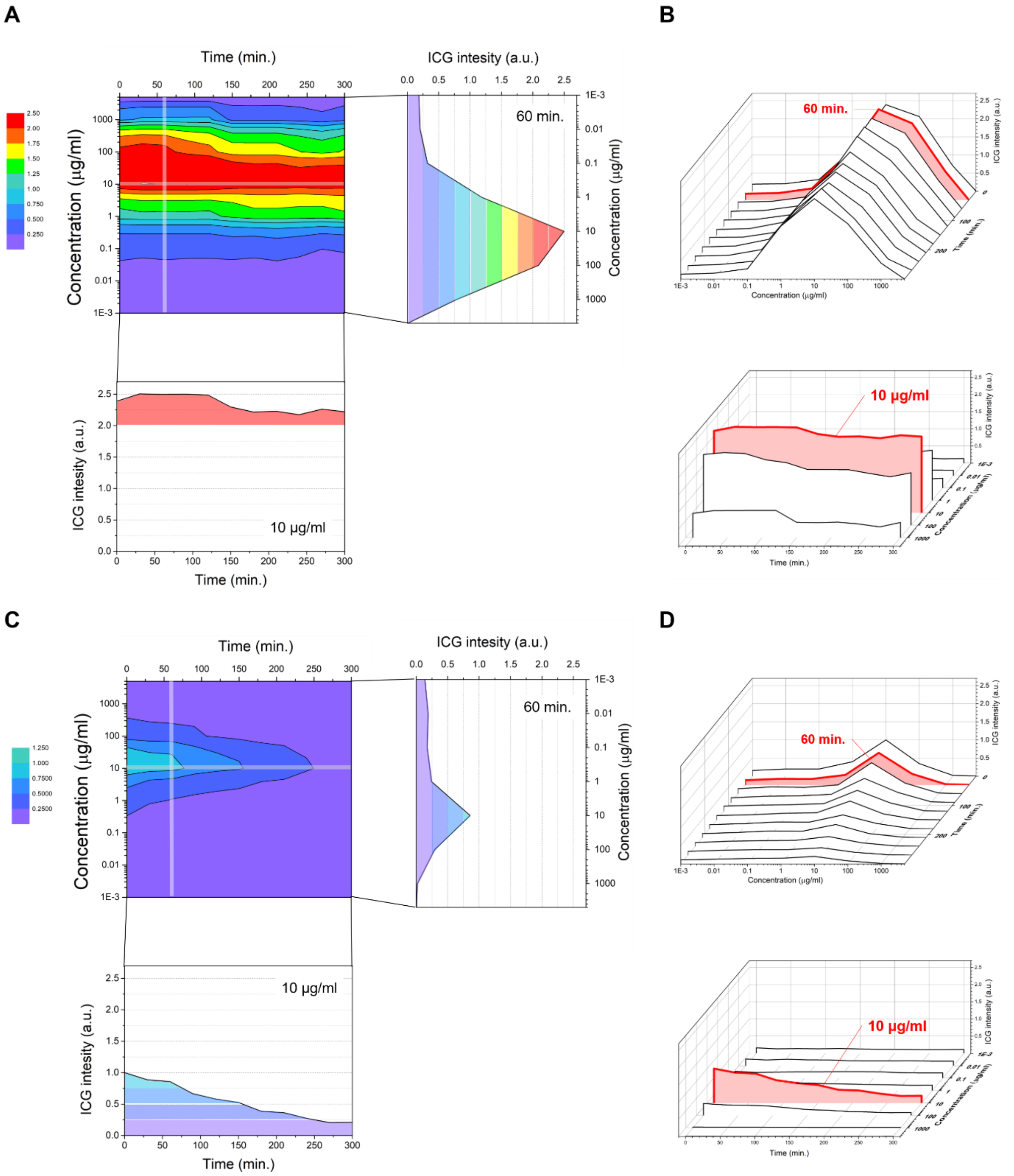

Supplementary Figure 1. (A) The rainbow colormap of in BSA solution in Figure 1, and the extracted graph for ICG intensity represented by a fixed concentration (10 µg/ml) over time (horizontal axis) and various concentrations at a fixed time point (60 min) (vertical axis). (B) The sequential visualization of ICG fluorescence changes in the waterfall plots at 60 min and 10 µg/ml, respectively. (C) The rainbow colormap of ICG intensity in distilled water solutions with corresponding concentration and time factors, and (D) its waterfall plots for distilled water solutions.

The cross-sectional profile of in vivo imaging for lymph nodes and lymphatic vessels

The cross-sectional profiles plotted from specific locations in the images allow the quantitative comparison of signal-to-noise ratios (SNR) and full width at half maximum (FWHM). As shown in Supplementary Figure 2, the anatomical locations of lymph nodes and lymphatic vessels in the images were identified using the color threshold function (blue color) in the ImageJ software after the injection of 0.05-mL Evans blue dye (30 mg/mL solution in 0.9% saline; Sigma, St Louis, MO). The cross-sectional profiles of the brachial lymph nodes (LNs; the yellow arrows) and collecting lymphatic vessels (LVs; the red arrows) were measured from the dot symbol along the direction of the dotted arrows. The dot symbol (starting point) and the direction of the dot arrows in Supplementary Figure 2A correspond to the same features in the graph in Supplementary Figure 2B. The FWHM in the graphs indicates that the LNs had a diameter of 1.96 mm, and the LVs had a diameter of 0.29 mm. The LNs and LVs identified in the visible images were represented as gray areas in Figure 5C. The graph of the cross-sectional profile in Figure 5 was measured using the plot profile function in ImageJ, following the same methodology in the visible image.

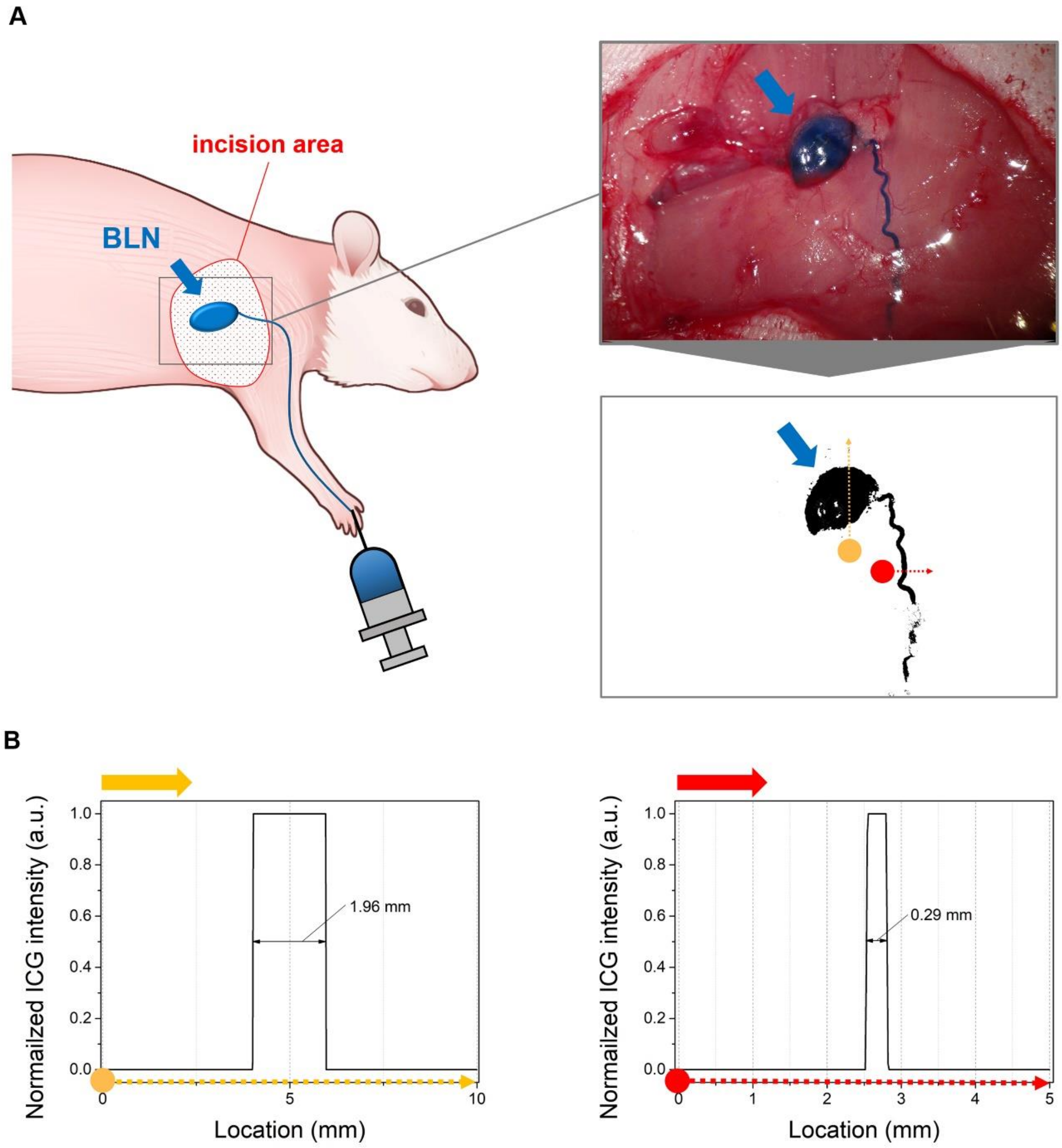

Supplementary Figure 2. (A) Method for obtaining the cross-sectional profiles from brachial lymph nodes (LNs) and collecting lymphatic vessels (LVs) following the injection of Evans blue. (B) The cross-sectional profiles of LNs (yellow) and LVs (red) were measured along the direction of the dotted arrow, starting from the dot symbols. Each graph's FWHM was respectively 1.96mm and 0.29mm.

NIRF imaging system

In this study, we used a customized NIRF imaging system for both in vitro and in vivo experiments. We represent actual photos of the system to aid in understanding (Suppl. Fig. 3). For accurate measurements, the imaging system features an adjustable stand that stabilizes the placement and distance between the light source and the detector. Designed for optimal performance, the device measures from 30 cm away from the subject, with a narrow depth of field of less than 2 mm. The lightsource was consistently delivered at intensity of 0.5 mW/mm^2 across the imaging area including region of interest (ROI).

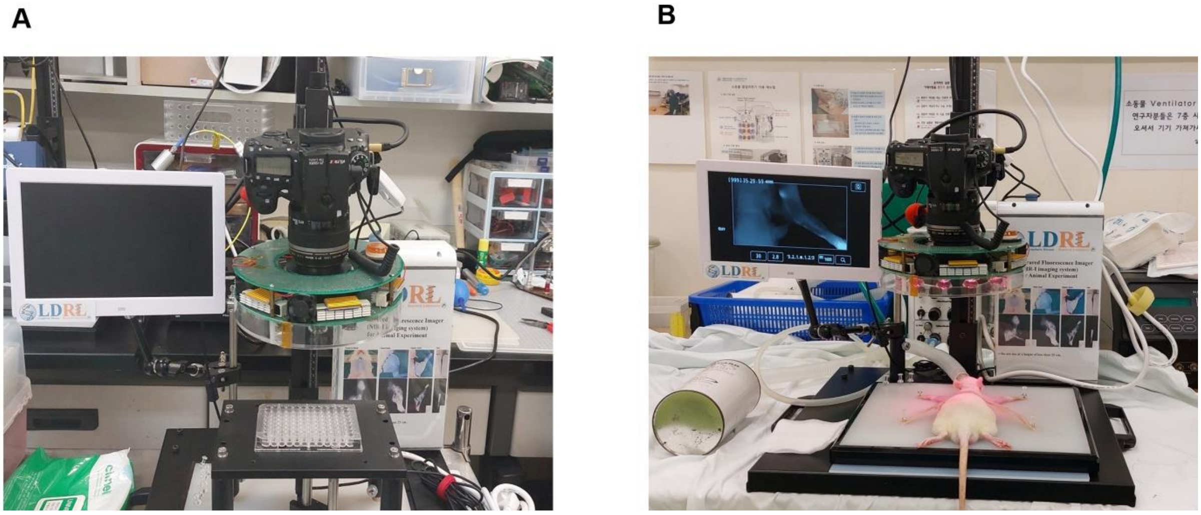

Supplementary Figure 3. The photos of the NIRF imaging system for the (A) in vitro and (B) in vivo experiments.
